# Putting ethics at the centre of health promotion practice: lessons from Australia

**DOI:** 10.1093/heapro/daaf205

**Published:** 2025-12-04

**Authors:** Krysten Blackford, Sharyn Burns, Jane Taylor, Francene Leaversuch, Tara Gamble, Gemma Crawford

**Affiliations:** Collaboration for Evidence, Research and Impact in Public Health (CERIPH), Curtin University, Boorloo, GPO Box U1987, Perth 6845, Western Australia; School of Population Health, Curtin University, Boorloo, Box U1987, Perth WA 6845, Australia; Collaboration for Evidence, Research and Impact in Public Health (CERIPH), Curtin University, Boorloo, GPO Box U1987, Perth 6845, Western Australia; School of Population Health, Curtin University, Boorloo, Box U1987, Perth WA 6845, Australia; School of Health, University of the Sunshine Coast, 90 Sippy Downs Drive, Sippy Downs 4556, Queensland; Collaboration for Evidence, Research and Impact in Public Health (CERIPH), Curtin University, Boorloo, GPO Box U1987, Perth 6845, Western Australia; School of Health, University of the Sunshine Coast, 90 Sippy Downs Drive, Sippy Downs 4556, Queensland; Collaboration for Evidence, Research and Impact in Public Health (CERIPH), Curtin University, Boorloo, GPO Box U1987, Perth 6845, Western Australia; School of Population Health, Curtin University, Boorloo, Box U1987, Perth WA 6845, Australia

**Keywords:** ethics, capacity building, competencies, evidence-informed practice, workforce development

## Abstract

This article examines recent developments to strengthen ethical health promotion practice in Australia and their global relevance. Ethical health promotion practice requires a dual foundation: structured ethical oversight combined with practitioners’ ability to implement ethical values and principles in their decision-making processes, leading to evidence-informed health promotion practice. However, health promotion organizations and practitioners report barriers to ethical practice, including limited resources and access to human research ethics committees and formal ethical oversight. Growing global recognition of inadequacies of traditional procedural approaches to ethical practice in health promotion highlights the need for initiatives and frameworks emphasizing decolonizing methodologies, relational and reflexive ethics, participatory and community-led oversight, and social justice embedded as a core principle. In response to barriers faced by health promotion practitioners and organizations in Australia, the Australian Health Promotion Association developed the Health Promotion Ethics Project to support ethical practice of the Australian health promotion workforce. A comprehensive ethics framework was developed comprising: (i) specialized ethics training and tools for health promotion practitioners; (ii) an ethical oversight committee with relevant expertise; (iii) customized submission processes and templates tailored to health promotion practice contexts; and (iv) advice to guide practitioners through ethical challenges. A pilot project to evaluate the framework was undertaken in 2024 with five diverse health promotion organizations across Australia, with findings informing several key recommendations for the global context. Continued investment in workforce development and tailored ethical oversight systems is vital for advancing the health promotion discipline and strengthening the global health promotion workforce.

Contribution to Health Promotion

- Positions ethics as a key component of everyday health promotion practice.
- Introduces a tailored ethics model developed by the Australian Health Promotion Association to support ethical review processes and practitioner decision-making through reflective, community-led approaches and access to formal ethical oversight.
- A pilot project undertaken with five organizations across Australia highlighted key components of the ethics model that could be adapted for other contexts.
- Lessons from Australia can help to further embed ethics into global health promotion efforts, supporting equity, social justice, and culturally safe practice.

## INTRODUCTION

Ethics is at the heart of health promotion practice, rooted in collective, social justice-oriented approaches to improving and optimizing population health and wellbeing (Carter *et al*. 2011, Carter 2014). Ethical health promotion practice relies on both formal ethical oversight and practitioners’ capacity to apply ethical values and principles to guide professional practice decisions and actions (Carter 2012, Blackford *et al*. 2022). The integration of ethics into daily practice is recognized internationally as a core competency for health promotion practitioners (Battel-Kirk *et al*. 2024). However, Australian evidence suggests practitioners encounter barriers to accessing appropriate ethical oversight mechanisms and resources that acknowledge the unique context and approaches of health promotion work (Reilly *et al*. 2016, Blackford *et al*. 2022). This article examines recent developments in Australia to strengthen ethical health promotion practice and their relevance in a global context.

## EVOLVING ETHICS IN HEALTH PROMOTION

Health promotion has undergone significant transformation in its approach to ethical considerations over the past four decades. However, there are ongoing tensions in the literature about the nature of ethics in health promotion practice. Whilst the origins of discourse about ethics in health promotion may be linked to the Ottawa Charter (World Health Organization 1986), it can also be traced back to more ancient philosophical traditions. Tountas (2009), e.g. highlighted changes in views towards health from ‘supernatural conceptions’ (p. 185) to those focused on broader sociopolitical determinants of health. This historical turn reflects the more critical perspective of health promotion, which rejects a wholly bio-behaviourist viewpoint and seeks a return to more holistic approaches. These tensions are reflected in early work by Manciaux *et al*. (1987), who argued that ‘people's health is very closely linked with their working and living conditions—more often imposed than freely chosen…’ (p. 1) highlighting the structural constraints that challenge individual responsibility narratives. Draper (1989) subsequently noted that consideration of policies to address health inequities requires consideration of both ethics and practicality. This foundational work called for developing a health promotion ethic, suggesting that ‘if there is any domain in which the ‘public’ should have a word to say (and more than a word) in putting together a social ethic, it is this one’ (Manciaux *et al*. 1987). Two decades later, Mittelmark (2007) observed that while health promotion possessed an ethical cornerstone in the Ottawa Charter, ‘an ethic for practice has yet to be codified’ (p. 84). These prescient observations laid the groundwork for subsequent decades of inquiry, and contemporary commitments to relational, reflexive and community-led ethics in health promotion.

## CHALLENGES FOR ETHICAL HEALTH PROMOTION PRACTICE

It is important to distinguish between ethics for health promotion research and ethics in health promotion practice, though boundaries can be blurred. Much health promotion practice, including programme delivery and community engagement processes, would not require Human Research Ethics Committee (HREC) approval as these areas of practice do not meet the definition of research involving human subjects. However, community assessment, programme evaluations, service innovations, co-design initiatives, and validation studies for tools, frameworks, or models require nuanced judgement, particularly where findings are to be generalized. As Thurston *et al*. (2003) argued, ‘when the objective…is to establish the effectiveness of the intervention, then the participants are research subjects who must give informed consent to an unproven intervention’ (p. 46). Similarly, Sainsbury (2015) contended that most action in health promotion involves humans and should be ‘bound by similar ethical principles and similar requirements for ethics review’ (p. 177) regardless of categorization as research, programme intervention, or evaluation.

Ethical health promotion requires both ethical oversight for health promotion research combined with practitioners’ ability to implement ethical values and principles in their decision-making processes, leading to evidence-informed practice (Carter 2012, Blackford *et al*. 2022). However, health promotion organizations and practitioners report barriers to ethical practice, particularly in the Australian context. Our research with Australian health promotion practitioners and organizations over the past decade highlights that many practitioners reflect a narrow conception of ethics as only obtaining formal oversight for research from a HREC, rather than in the role of active engagement in ongoing and reflexive ethical practice (Reilly *et al*. 2016, Blackford *et al*. 2022). Additionally, many health promotion organizations report having limited access to HRECs, making formal ethical oversight challenging (Reilly *et al*. 2016, Blackford *et al*. 2022). Practitioners have also identified other barriers to ethical practice including limited resources, lack of support for preparing ethics applications, and concerns that ethics approval is not seen as core business by their organization (Reilly *et al*. 2016).

Health promotion operates beyond traditional academic and clinical health settings, highlighting the need to ensure ethics application and review processes employ approaches and methods that extend beyond those reflected in the biomedical model (Carter 2014). While historical ethical review frameworks are rooted in biomedical paradigms that can be insufficient for addressing population-level and equity-driven challenges of health promotion practice (Carter 2012, 2014; Carter *et al*. 2011, McPhail-Bell *et al*. 2015), new challenges have been recognized, including the use of participatory methods and working with diverse populations (Smyth *et al*. 2024, National Health and Medical Research Council 2025). Formal ethical oversight mechanisms have been criticized for failing to accommodate the realities of health promotion work at the community level, leading to tensions between procedural requirements and culturally safe practices (Pyett *et al*. 2008, Haintz *et al*. 2015, McPhail-Bell *et al*. 2015). When these tensions exist, health promotion practitioners face ongoing challenges when navigating between institutional rules, formal ethical review frameworks, and the lived experiences of priority populations (Pyett *et al*. 2008, Haintz *et al*. 2015, D’souza *et al*. 2018). Relational and participatory approaches are key to ethical health promotion work, grounded in reflexivity, power sharing, and responsiveness to community priorities rather than focusing solely on individual consent and minimizing risk of harm (Carter 2012, 2014; Carter *et al*. 2011, Tretheway *et al*. 2015). However, traditional ethical oversight mechanisms have been criticized for inadequately addressing issues of equity, power dynamics, and meaningful community participation that are central to health promotion’s mandate (Carter 2014, McPhail-Bell *et al*. 2015).

The limitations of traditional ethical frameworks for health promotion extend beyond access issues to fundamental paradigmatic concerns. Azétsop and Rennie (2010) suggested that ‘the medical individualism underlying autonomy-based bioethics renders the latter incapable of addressing some of the most pressing bioethical issues in resource-poor settings, which have to do with social justice’ (p. 1). This critique is supported by Sindall (2002) who questioned whether more macro approaches were required, beyond principles of bioethics to incorporate theories from social and political philosophy. These challenges are amplified in cultural contexts, particularly regarding Indigenous populations. McPhail-Bell *et al*. (2015) argued that little attention has been paid to the colonial basis of health promotion with its foundations in the Ottawa Charter (World Health Organization 1986), leading to policies and programmes founded upon a paternalistic logic. The persistence of Western-centric approaches to health promotion ethics suggests a failure to decolonize both theory and practice.

The evolution of health promotion has revealed tensions between different ways of knowing that carry significant ethical implications. Health promotion practice encompasses diverse activities beyond programme delivery and community engagement, including policy advocacy, systems change, and intersectoral action, each posing distinct ethical challenges. As Cribb (2015) noted, multisectoral work requires diplomatic ethics in interdisciplinary contexts, with practitioners needing to be ‘bilingual, or better multilingual, about values and can recognize the philosophical boundaries they work across’ (p. 203). Potvin and Jourdan (2021) identified a ‘potluck model’ (p. 26) where researchers from various disciplines apply their methods without sufficient integration, creating ethical consequences as different epistemological approaches embody different assumptions about knowledge, participation, and social change. Cross *et al*. (2024) suggest that generating evidence from randomized controlled trials may present ethical and practical challenges and contradictory to the underpinning values of health promotion. This tension is particularly evident in Indigenous research contexts, where Pyett *et al*. (2008) argue for decolonizing approaches that privilege ‘reciprocity, respect, and capacity exchange’ with focus on ‘values, processes and relationships’ (p. 181). Klinner *et al*. (2014) found Australian practitioners struggling to integrate relationship-based and research-based approaches, with practitioners reporting frustrations regarding research that was ‘insufficient or not transferable to the work that participants wanted to do with communities’ (p. 895).

## ADDRESSING BARRIERS TO ETHICAL PRACTICE

Global initiatives aimed at overcoming barriers to ethical health promotion practice are situated within broader movements towards decolonization, social justice, and participatory practice (World Health Organization 2016, Hawks *et al*. 2023). A key criticism of dominant ethical frameworks has been their connection to colonial and imperialist traditions that may perpetuate harmful power dynamics and fail to meet the needs of priority groups in various contexts (D’souza *et al*. 2018, Chandanabhumma and Narasimhan 2020, Naidu *et al*. 2024). Researchers in diverse global settings have called for shifting from procedural, rule-bound ethical oversight to ongoing reflexivity and negotiated relationships with communities, emphasizing moving ethical decision-making towards practice-based processes while reflecting critically on power, positionality, and local accountability (D’souza *et al*. 2018, Naidu *et al*. 2024). e.g. health promotion work with Indigenous communities is increasingly engaging with Indigenous concepts of data sovereignty and self-determination, highlighting the need for community-controlled ethical review mechanisms and participatory governance models (Pyett *et al*. 2008, Chandanabhumma and Narasimhan 2020, Naidu *et al*. 2024). Achieving health equity and redressing the legacy of colonial and structural injustices are core ethical imperatives for health promotion (Crouch and Fagan 2014, Chandanabhumma and Narasimhan 2020, Naidu *et al*. 2024).

Examples of ethical frameworks and approaches that prioritize community participation and control can be found within the Australian context. The AIATSIS Code of Ethics for Aboriginal and Torres Strait Islander Research emphasize the importance of acknowledging and respecting Indigenous peoples’ rights, ensuring that Aboriginal and Torres Strait Islander communities are actively engaged in any processes, projects, or activities that may impact them (Hawks *et al*. 2023). This commitment is operationalized through mechanisms like the Western Australian Aboriginal Health Ethics Committee and regional planning forums in Western Australia, which maintain community participation, decision-making, and control over research and programmes involving Aboriginal people. Similarly, the ACON Ethics Committee provides an important example of community-led ethical oversight, supporting research and programmes that involve LGBTQ+ communities to ensure that these are conducted in ways that uphold community rights, priorities, and wellbeing (ACON 2025) and prioritize principles of meaningful involvement stemming from the human immunodeficiency virus (HIV) response (Global Network of People Living with HIV/AIDS (GNP+) and Asia Pacific Network of People Living with HIV/AIDS (APN+) 2016). Other examples comprising volunteer experts responsible for the review, oversight and monitoring of research include the RACGP National Research and Evaluation Ethics Committee (The Royal Australian College of General Practitioners (RACGP) 2025) and the Cancer Council Victoria HREC (Research Governance Unit 2025).

Beyond Australia, examples of community-level oversight mechanisms can be found in Canada and New Zealand, demonstrating how alternative approaches can remain responsive to local contexts and needs. The Aotearoa Research Ethics Committee (Aotearoa Research Ethics Committee 2025) (formerly the New Zealand Ethics Committee) provides independent ethical review for community, professional, and government researchers in New Zealand who cannot access institutional ethics committees, emphasizing voluntary participation and respect for researcher autonomy alongside participant rights. The Community Research Ethics Office (Community Research Ethics Office 2025) in Canada focuses on strengthening community-based research through accessible ethics support, following the Tri-Council Policy Statement (Government of Canada 2022) and First Nations OCAP principles (ownership, control, access, and possession) (First Nations Information Governance Centre n.d.), with a strong emphasis on stakeholder engagement, equity, and the Truth and Reconciliation Commission recommendations (Truth and Reconciliation Commission of Canada 2015). Meanwhile, the Human Research Ethics Board of Alberta Community Health Committee (Health Research Ethics Board of Alberta 2025) provides oversight for community-based investigators and organizations in Alberta, covering both health and non-health research, as well as higher-risk non-research projects. Common design features, including accessibility beyond institutions, regular cycles, cost sensitivity, and attention to Indigenous principles and community governance, offer practical lessons for a health-promotion-aligned model while maintaining rigour tailored to community contexts. These developments are consistent with broader calls to move from proceduralism to reflexive, participatory, decolonizing ethics attentive to power and justice (Carter *et al*. 2011, Chandanabhumma and Narasimhan 2020, Naidu *et al*. 2024).

The growing global recognition of the inadequacies of traditional procedural approaches to ethical practice in health promotion highlights the need for initiatives and frameworks emphasizing decolonizing methodologies, relational and reflexive ethics, participatory and community-led oversight, and social justice embedded as a core principle. Several Australian studies have highlighted the need for frameworks and professional resources to support everyday decision-making and reflective ethical values and practice for health promotion practitioners (Axford and Carter 2015, Reilly *et al*. 2016, Blackford *et al*. 2022). However, there is no consensus on a code of ethics for health promotion practice at the national or global level. Similarly, there is a paucity of empirical evaluations of community-based ethical review mechanisms and few concrete recommendations for adapting ethical frameworks to health promotion accreditation or registration systems reported in the literature. Strategies to address the challenges in translating key values into practical mechanisms for ethical health promotion practice, governance, and professional accreditation are vital to strengthen the health promotion discipline and profession.

## THE AUSTRALIAN RESPONSE

In response to the barriers faced by health promotion practitioners and organizations, the Australian Health Promotion Association (AHPA) developed the Health Promotion Ethics Project (HPEP) to support the ethical practice of the Australian health promotion workforce (Australian Health Promotion Association 2023, Blackford *et al*. 2025). AHPA is Australia’s professional association dedicated to the practice, policy, research, and study of health promotion, and committed to ethical practice via ‘culturally informed, participatory, respectful and safe practice’ (Australian Health Promotion Association 2020). HPEP was developed to provide opportunities to build AHPA’s capacity to lead conversations about ethical health promotion practice; and aims to facilitate access to formal HREC support for Australian health promotion evaluation and research. Building on the formative research conducted with Australian practitioners and organizations over the past decade (Reilly *et al*. 2016, Blackford *et al*. 2022), and informed by international community-level ethics oversight mechanisms (Aotearoa Research Ethics Committee 2025, Community Research Ethics Office 2025, Health Research Ethics Board of Alberta 2025), a comprehensive ethics framework was developed comprising: (i) specialized ethics training and tools for health promotion practitioners; (ii) a dedicated ethical oversight committee with relevant expertise; (iii) customized submission processes and templates tailored to health promotion contexts; and (iv) advice to guide practitioners through ethical challenges.

The HPEP ethics framework was designed as an enabling mechanism that complements (rather than replaces) institutional research governance. Its objectives were to build workforce capabilities for ethical reflection and decision-making; provide discipline-relevant ethical advice for practice, evaluation and research proposals; tailor documentation to health promotion contexts; and support navigation of formal HREC pathways where required. The model’s ethical advice process does not issue formal HREC approvals; rather, it provides readiness review and practical guidance aligned with existing frameworks.

### Pilot project

In 2024, five organizations without routine access to HRECs were purposively recruited to reflect diverse settings (charitable, community health service, consultancy, and advocacy). Organizations selected lacked HREC access yet demonstrated a commitment to enhancing their staff's capacity for ethical health promotion practice. Each submitted a prospective project using adapted, health promotion specific templates via the National Health and Medical Research Council’s Human Research Ethics Application (HREA) portal. Ethical advice was provided by reviewers with health promotion research expertise, aligning feedback with the National Statement on Ethical Conduct in Human Research (2023) (National Health and Medical Research Council 2023) and health promotion values and principles outlined in the Core Competencies and Professional Standards for Health Promotion (International Union for Health Promotion and Education 2016). Participants received support materials, including application instructions, protocol templates, and participant information and consent form templates; additional application support was available.

Evaluation of the pilot project provided insights on the acceptability, appropriateness, and usability of the model (Saunders *et al*. 2005). The evaluation employed mixed methods comprising HPEP observations and notes, online surveys, and semi-structured interviews with participants, which were analysed to identify recurrent themes related to these elements. The study was approved by the Curtin University Human Research Ethics Committee (HRE2018-0523).

The pilot evaluation of the HPEP provides valuable insights into the practicalities and challenges of embedding ethical oversight within the Australian health promotion sector. Participants consistently valued the simplified, health-promotion-aligned pathway offered by the HPEP model. However, the requirement to use the HREA portal reintroduced biomedical terminology and workflows that felt incongruent with community-based work. As one participant noted, ‘…the main benefit…was all negated a bit when I still had to use the [traditional] form’. This highlights the ongoing tension between traditional ethical review frameworks rooted in biomedical paradigms, and the realities of health promotion practice, which often require more relational and context-sensitive approaches (Carter 2014, McPhail-Bell *et al*. 2015).

A key theme emerging from the pilot was the need for practical support and mentoring to build ethical reasoning capabilities within organizations. Rather than focusing solely on forms and templates, participants emphasized that ‘it’s more the skill set and knowledge of people who are expected to use it than the platform itself’. This finding aligns with previous research underscoring the importance of workforce development and capacity building in ethical reflection and decision-making (Axford and Carter 2015, Tretheway *et al*. 2015).

The value of discipline-aligned, practical feedback was also evident. Review by health promotion experts was rated highly, with participants describing the feedback as ‘really targeted…sensible and practical’. This suggests that ethical review processes tailored to the specific context and values of health promotion can enhance both the relevance and acceptability of ethical oversight.

Importantly, the pilot process supported a cultural shift towards ethical deliberation and readiness for dissemination. One participant reflected on ‘changing the culture…slowing down and doing these things properly’, indicating that the model fostered a more reflective approach to ethical practice. This cultural shift is critical for embedding ethics as a core component of everyday health promotion work, moving beyond compliance to genuine engagement with ethical principles (Parker *et al*. 2007, Carter *et al*. 2011).

Despite these positive outcomes, several limitations must be acknowledged. The pilot involved only five organizations, introducing potential self-selection bias, and longer-term evaluation is needed to assess sustained impacts. The advice process did not constitute formal HREC approval, and the use of the HREA portal may have inadvertently reinforced biomedical workflows. Additionally, the model primarily addressed programme delivery and community engagement, suggesting a need for further adaptation to support policy advocacy and systems change. Future scale-up will require clear governance, conflict-of-interest management, reviewer training, and evaluation of downstream outcomes. Research should also explore integration into accreditation systems and effectiveness across diverse cultural contexts.

## GLOBAL IMPLICATIONS

The Australian model offers several lessons for global health promotion workforce development. Key components such as community-accessible review processes, health-promotion-specific templates, reviewer panels with practice expertise, clear HREC referral pathways, resource-sensitive fee structures, and integration with workforce development could be adapted for other contexts. These features address many of the barriers identified in the literature, including limited access to formal ethical oversight, lack of tailored resources, and the need for discipline-specific support (Reilly *et al*. 2016, Blackford *et al*. 2022).

Specialized training is required to move practitioners beyond narrow understandings of ethics reported in the literature, towards capacity building in critical reflection and ethical decision-making (Axford and Carter 2015, Tretheway *et al*. 2015, Reilly *et al*. 2016, Blackford *et al*. 2022). This may be achieved through formal education, peer support, and mentoring, e.g. via communities of practice. A skilled, confident workforce will be better equipped to navigate the complex and context-specific ethical issues that arise in health promotion work globally (Axford and Carter 2015, Blackford *et al*. 2022). Further, the ethical application process usually strengthens health promotion projects and endeavours, affording more protection for participants and communities as well as for practitioners and organizations (Smyth *et al*. 2024).

Lessons from Australia underscore the role of professional health promotion associations in developing and implementing initiatives to support meaningful systems change. We recommend other professional health promotion associations supporting the development of specialized community-led ethical review mechanisms specifically tailored to health promotion practice, alongside significant investment in workforce capacity building around ethical decision-making. Association-led ethical review mechanisms may offer alternatives to traditional institutional oversight, supporting discipline-embedded processes and procedures to deal with the unique requirements of health promotion practice (Blackford *et al*. 2022). This includes specific support resources and reviews conducted by association members with expertise in health promotion ethical practice. Ethical oversight processes should be robust [e.g. the national statement highlights the importance of sufficient time for reviews (National Health and Medical Research Council 2025)] yet not prohibitive for community-based practitioners and organizations working with limited resources and within prescriptive funding deadlines, to address any gaps in institutional oversight access or other barriers previously reported in the literature (Reilly *et al*. 2016, Blackford *et al*. 2022). Professional health promotion associations should consider establishing partnerships with existing community-based ethics committees where available or developing new mechanisms following principles established in Canada and New Zealand. The IUHPE could facilitate knowledge sharing between national associations to support broader implementation of such models globally. Scale-up requires sustainable funding models, possibly through membership fees, sliding scale structures for community organizations, or government support recognizing the public health value of ethical health promotion practice.

Strengthening the health promotion evidence-base by increasing the dissemination of health promotion evaluation and case studies in the peer-reviewed literature is critical (Reilly *et al*. 2016, Blackford *et al*. 2022). This can be achieved via increased access to ethical oversight mechanisms and ethical practice capacity building initiatives, which may support enhanced research and evaluation capacity in the global health promotion sector. We also recognize the importance of working with journals to better support the publication of community-based health promotion research, including studies conducted with strong community support but outside traditional ethics oversight structures. There is also an opportunity to further evaluate and refine the ethical values and principles embedded into accreditation and professional standards to enhance the professional identity of the workforce.

## THE IMPERATIVE FOR ONGOING ETHICAL OVERSIGHT AND REFLEXIVE PRACTICE

Developments and scholarship over the past 40 years reveal both the complexity of health promotion ethics and the urgent need for more critical engagement with and development of more theoretical frameworks. The shift from procedural to relational, reflexive ethical approaches requires explicit attention to power dynamics and value commitments. As Sindall (2002) argued, health promotion can ‘no longer take its own moral credentials for granted’ (p. 203). Parker *et al*. (2007) emphasized that practitioners must be cognisant and reflective of their philosophical and moral foundations and how these relate to those of the communities with which they work. Tretheway *et al*. (2015) identified critical reflection as a missing ethical competency that provides a mechanism for health promotion practitioners to explicitly attend to ethical values and principles. This reflexive turn acknowledges that health promotion cannot claim neutral positions but must engage with the power dynamics inherent in practice. As Carter (2012) noted, ‘health promotion is a moral project…that…can be practised in ways that are more or less ethical’ (p. 4). The field’s ethical future lies in cultivating capacities for critical analysis, reflexive practice, and transformative action that can respond to complex challenges of promoting health in an inequitable world. This includes developing what Bentley (2013) termed an ecological public health model with principles of conviviality, equity, sustainability, and global responsibility that acknowledge our place within, rather than dominance over, ecosystems.

While health promotion’s moral authority may assert a prima facie ethical stance (McPhail-Bell *et al*. 2015), the literature challenges assumptions about health promotion’s inherent ‘rightness’. Gillon (1990) called for greater scrutiny of assumptions regarding obvious benefits of health promotion, with recommendations for oversight of interventions. More provocatively, Gugglberger (2018) asked, ‘what if some interventions do not only do good, what if they—unintentionally—cause harm?’ (p. 557). Similarly, Greyson *et al*. (2018) challenged assumptions that information interventions are non-invasive and ethically innocuous. These assertions reinforce the need for continued engagement with formal ethical oversight mechanisms. Allen and Flack's (2015) insider perspective as HREC members offered practical insights, suggesting that regardless of how activities are categorized, e.g. research, quality assurance, or evaluation, the activity is using people for one’s own ends, rather than for their benefit, advocating for careful consideration of ethical responsibilities.

Future developments in health promotion ethics must grapple with emerging challenges including climate change and intergenerational justice. Bentley's (2013) ecological public health model proposed principles of conviviality, equity, sustainability, and global responsibility that acknowledge humans as part of, and not separate from ecosystems. As Potvin (2025) identified global trends towards widening income inequality and decreasing social inclusion, ‘we must now work to change the rules of the game, ensuring that the rules of social cohesion that are currently being defined do not become entrenched and shape our future’ (p. 5). In the context of our call for action to institute and operationalize an agenda for health promotion ethics in Australia and beyond, we reiterate critiques from McPhail *et al*. (2015) who have rightly pointed out that the lack of critical ethical reflection and accountability may risk stigmatization and the reinforcement of health inequality. This perspective represents a shift beyond traditional approaches, suggesting that future health promotion ethics must address questions traditional frameworks have not adequately considered.

## CONCLUSION

The Australian experience highlights the challenges and opportunities for developing reflexive, contextually relevant, and participatory ethical oversight and capacity building mechanisms for health promotion practitioners and organizations. By addressing the distinct needs of health promotion and the communities that benefit from its actions, professional health promotion associations can support more effective, accountable, and equitable practice. Positioning ethical oversight as an enabler of reflective practice, rather than a compliance hurdle, appears central to feasibility and acceptability for community-based health promotion. Continued investment in workforce development and tailored ethical oversight systems is vital for advancing the health promotion discipline and strengthening professional identity of the global health promotion workforce. Moving forward, we assert that health promotion, both as a profession and a discipline, requires a reflexive turn, acknowledging that health promotion cannot claim neutral or objective positions but must explicitly engage with the power dynamics and value commitments inherent in all practice. The field’s ethical future lies not only in developing universal principles but in cultivating capacities for critical analysis, reflexive practice, and transformative action that can respond to the complex and evolving challenges of promoting health in an inequitable world.

## Data Availability

The data underlying this article cannot be shared publicly due to protection of the privacy of individuals and organizations that participated in the study. The data will be shared on reasonable request to the corresponding author.
